# Gastric mucormycosis

**DOI:** 10.4322/acr.2023.421

**Published:** 2023-02-10

**Authors:** Sourav Bhowmik, Devendra Jadav, Divya Aggarwal, Raghvendra Singh Shekhawat

**Affiliations:** 1 All India Institute of Medical Sciences, Department of Forensic Medicine and Toxicology, Jodhpur, Rajasthan, India; 2 All India Institute of Medical Sciences, Department of Pathology and Lab Medicine, Jodhpur, Rajasthan, India

**Keywords:** Mucormycosis, Diagnosis, Autopsy, Pathology, Pyloric Antrum

Mucormycosis is a life-threatening fungal infection caused by mucormycetes, fungi of the Mucorales order.^1^ Rhino-orbital-cerebral involvement is the most common form of invasive mucormycosis. However, gastrointestinal (GI) mucormycosis cases have increased in the last two to three decades.^2^ Involvement of the GI tract in invasive mucormycosis is seen in 7–13% of cases.^3^ Out of which, involvement of the stomach is seen in 58% of the cases, and the remaining 42% involve small and large intestines. GI mucormycosis has been mostly associated with immunocompromised patients or premature infants.^2^ It has also been reported in immunocompetent patients.^1^ Many cases of GI mucormycosis are first recognized on autopsy, owing to its acute course and rapidly fatal nature.^2^ According to the literature, only about 25% of cases of GI mucormycosis are clinically diagnosed.^4^

**Figure 1:**
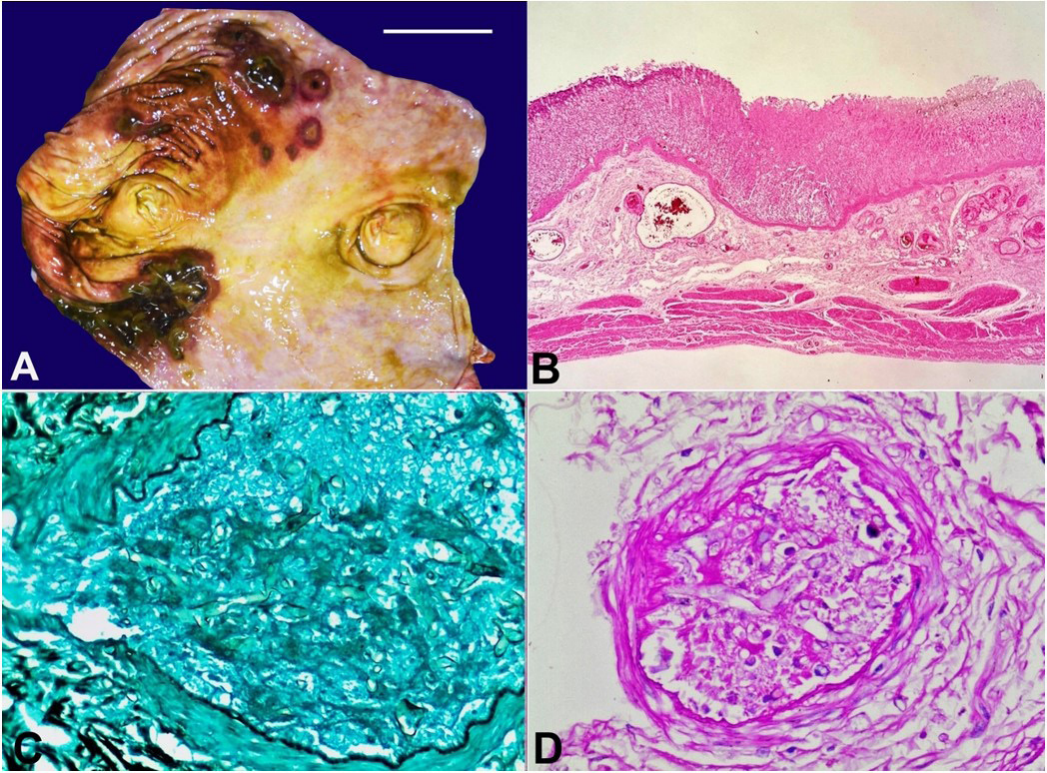
**A** - Macroscopic view of the stomach with multiple targetoid lesions of varying size, round to oval in shape with elevated margins and necrotic base (scale bar= 5 cm); **B** - Section from stomach depicting the transition zone between viable and necrotic tissue, left side of the image shows viable gastric tissue with patent submucosal blood vessels, while the right side shows bland necrosis with obliterated blood vessels (H&E, 20x); **C** and **D -** Gomori methenamine silver (GMS) and Periodic acid Schiff (PAS) stains highlight the angioinvasive fungal profiles within the submucosal blood vessels (400x). These hyphae are broad, aseptate foldable with right angle branching conforming to the morphology of mucormycosis.

The endoscopic appearance of gastric mucormycosis is usually a large ulcer with necrosis, eventually presenting an adherent, thick, green exudate.^3^ Figures 1A typically show green exudates surrounding the lesions. Autopsy diagnosis of GI mucormycosis is mostly based on gross and histopathological examination. Grossly, numerous well-circumscribed, dark red, targetoid mucosal lesions have been described in the post-mortem diagnosis of GI mucormycosis.^4^ Figures 1A show the so-called ‘targetoid lesion’ in the stomach mucosa.

Clinical diagnosis of GI mucormycosis is challenging. It can present an array of nonspecific symptoms, including discomfort, diarrhea, fever, gastrointestinal bleeding, necrosis, perforation, and as a necrotizing enterocolitis in premature neonates.^5^ The mechanism of GI tract involvement in mucormycosis is unclear. Pre-existing peptic ulcer disease, consumption of food and water contaminated with Mucorales, and use of contaminated nasogastric tubes, tongue depressors, and wooden spatula are a few factors responsible for the involvement of the GI system in mucormycosis.^5^^,^^6^ Mortality rate of GI mucormycosis is reported as 40 to 78%.^4^^,^^5^ Perforation of necrotic ulcers and peritonitis are the leading causes of death in such cases.

The reported images belong to a 48-year-old man who died from septicemia seven days after a road traffic accident. At autopsy, apart from findings of traumatic injuries, the stomach showed multiple rounds to oval lesions with sizes ranging from 0.5cm X 0.5 cm to 3cm X 2 cm, over the pylorus with elevated margins and necrotic base (Figure 1A). On the formalin-fixed specimen, they were noted as ulcers with a greenish-black base and flattened edges. The periphery of the ulcer was congested.

The microscopic examination showed that the ulcers were extending to the muscularis propria. They were invading underlying arteries and veins in the submucosa, indicating angioinvasion (Figure 1B). Gomori methenamine silver (GMS) and Periodic acid Schiff (PAS) stains highlighted fungal hyphae, which were broad aseptate and foldable, confirming the morphology of mucormycosis (Figure 1C and 1D, respectively).

In conclusion, gastric mucormycosis cases usually go unnoticed clinically unless it becomes symptomatic or diagnosed incidentally on endoscopy. Thus, such cases are being diagnosed at autopsy.
